# A randomized clinical trial on inhaled ciclesonide for managing acute asthma in the emergency room

**DOI:** 10.1590/1516-3180.2021.0542.R1.15092021

**Published:** 2022-04-11

**Authors:** Demétrius Tierno Martins, Karla Carlos, Luciane Bizari Carvalho, Lucila Bizari Prado, Carolina Fransolin, Alvaro Nagib Atallah, Gilmar Fernandes do Prado

**Affiliations:** I MD. Physician, Department of Emergency Medicine and Evidence-Based Medicine, Escola Paulista de Medicina (EPM), Universidade Federal de São Paulo (UNIFESP), São Paulo (SP), Brazil.; II PhD. Physiotherapist, Neuro-Sono Sleep Center, Department of Neurology, Escola Paulista de Medicina (EPM), Universidade Federal de São Paulo (UNIFESP), São Paulo (SP), Brazil.; III PhD. Psychologist, Neuro-Sono Sleep Center, Department of Neurology, Escola Paulista de Medicina (EPM), Universidade Federal de São Paulo (UNIFESP), São Paulo (SP), Brazil.; IV MD. Physician, Neuro-Sono Sleep Center, Department of Neurology, Escola Paulista de Medicina (EPM), Universidade Federal de São Paulo (UNIFESP), São Paulo (SP), Brazil.; V MSc. Physiotherapist, Neuro-Sono Sleep Center, Department of Neurology, Escola Paulista de Medicina (EPM), Universidade Federal de São Paulo (UNIFESP), São Paulo (SP), Brazil.; VI MD, PhD. Professor, Department of Emergency Medicine and Evidence-Based Escola Paulista de Medicina (EPM), Universidade Federal de São Paulo (UNIFESP), São Paulo (SP), Brazil.; VII MD, PhD. Professor, Neuro-Sono Sleep Center, Department of Neurology, Escola Paulista de Medicina (EPM), Universidade Federal de São Paulo (UNIFESP), São Paulo (SP), Brazil.

**Keywords:** Clinical trial as topic, Emergency service, hospital, Therapeutics, Bronchial asthma, Treatment, Room, emergency

## Abstract

**BACKGROUND:**

Use of inhaled corticosteroids for managing acute asthma exacerbations has been tested since the 1990s.

**OBJECTIVE:**

To compare high doses of inhaled ciclesonide with systemic hydrocortisone for managing acute asthma exacerbations in the emergency department.

**DESIGN AND SETTING:**

Double-blind, randomized clinical trial in the public healthcare system of the city of São Paulo.

**METHODS:**

Fifty-eight patients with moderate or severe asthma with peak flow < 50% of predicted were randomized into two groups. Over the course of four hours, one group received 1440 mcg of inhaled ciclesonide plus hydrocortisone-identical placebo (ciclesonide + placebo), while the other received 500 mg of intravenous hydrocortisone plus ciclesonide-identical placebo (hydrocortisone + placebo). Both groups received short-acting bronchodilators (fenoterol hydrobromide and ipratropium bromide). The research protocol included spirometry, clinical evaluation, vital signs and electrocardiogram monitoring. Data were obtained at 30 (baseline), 60, 90, 120, 180, and 240 minutes. We compared data from baseline to hour 4, between and within groups.

**RESULTS:**

Overall, 31 patients received ciclesonide + placebo and 27 received hydrocortisone + placebo. Inhaled ciclesonide was as effective as intravenous hydrocortisone for improving clinical parameters (Borg-scored dyspnea, P = 0.95; sternocleidomastoid muscle use, P = 0.55; wheezing, P = 0.55; respiratory effort, P = 0.95); and spirometric parameters (forced vital capacity, P = 0.50; forced expiratory volume in the first second, P = 0.83; peak expiratory flow, P = 0.51).

**CONCLUSIONS:**

Inhaled ciclesonide was not inferior to systemic hydrocortisone for managing acute asthma exacerbations, and it improved both clinical and spirometric parameters.

**TRIAL REGISTRATION:**

RBR-6XWC26 - Registro Brasileiro de Ensaios Clínicos (http://www.ensaiosclinicos.gov.br/rg/RBR-6xwc26/).

## INTRODUCTION

Asthma is a chronic inflammatory disease of the airways that affects approximately 300 million people worldwide. In the United States, between 2001 and 2003, asthma exacerbations caused 4,210 deaths, 504,000 hospitalizations and 1.8 million emergency room visits.^1^

Since the mid-1990s, inhaled corticosteroids have been tested for managing asthma exacerbations in emergency-room settings. They have systemic corticosteroid-sparing potential and avoid the need for venipuncture, which is sometimes a difficult procedure.^2-4^ These drugs exert vasoconstrictor effects on the mucosa by reducing neuronal reuptake of noradrenaline at the neuromuscular junctions of mucosal vessels, thus reducing secretions and facilitating the delivery of beta-2 agonists to their target receptors. Their onset of action is rapid, with peak vasoconstriction occurring in 30 minutes and lasting up to 90 minutes after inhalation.^5,6^

Ciclesonide is a prodrug that is activated at the site of action (bronchial cells and lining fluid of the bronchus) by bronchial esterases. These convert ciclesonide to desisobutyryl ciclesonide, which has 100-fold greater affinity for the glucocorticoid receptor than ciclesonide itself.^7^ Because of this peculiar property, common side effects such as hoarseness, dysphonia, oral candidiasis and suppression of the hypothalamic-pituitary-adrenal axis are much less frequent with ciclesonide than with other high-dose inhaled corticosteroids, as it is inactive outside the lung.^7-11^

The anti-inflammatory action of intravenous corticosteroids occurs via a genomic mechanism. This reduces expression of proinflammatory mediators such as interleukins^12^ and upregulates expression of beta-adrenoceptors in bronchial smooth muscle tissue. This effect is also shared by inhaled corticosteroids and begins four to six hours after administration,^13^ although some studies have shown that systemic corticosteroids administered to severely ill patients up to one hour after emergency department admission yields clinical benefits, such as reduced hospitalization rate and shorter length of emergency department stays.^14,15^ Data from double-blind randomized controlled trials have suggested that, compared with systemic corticosteroids, inhaled corticosteroids can decrease admission rates and allow earlier discharge from the emergency department. Peak flow levels and forced expiratory volume in the first second (FEV1) also rise more quickly in patients who are given inhaled corticosteroids.^16-19^

## OBJECTIVE

To the best of our knowledge, this was the first double-blind randomized clinical trial with the objective of comparing high doses of inhaled ciclesonide with use of injectable hydrocortisone for managing acute asthma in emergency settings. This trial was justified by the expected potential for fewer side effects with inhaled ciclesonide, the supposed benefit of its rapid onset of action and the need for more inhaled drugs to be available for clinicians dealing with asthma exacerbation in the emergency department.

## METHODS

### Population and setting

We studied patients with asthma aged 13 years or older, of both sexes, in the city of São Paulo, Brazil. Patients were recruited from the emergency department of Hospital São Paulo (a teaching hospital that is part of the Universidade Federal de São Paulo [UNIFESP]) and from two freestanding public urgent care centers affiliated with the hospital: Assistência Médica Ambulatorial (AMA) Santa Cruz and AMA Sacomã.

We included patients with a previous diagnosis of asthma (dyspnea, coughing, wheezing and chest tightness, associated with allergen exposure or cold air)^20,21^ who received follow-up at outpatient clinics within the catchment area of the Hospital São Paulo emergency department and had a peak flow < 50% of the predicted flow. All participants had a longstanding history of asthma, with repeated exacerbations and emergency room visits. The patients who we included had had at least two years of moderate or severe asthma, with a mean peak flow immediately before intervention of 163 liters/min.

We excluded patients with body temperature ≥ 37.8 °C, smokers, pregnant women, patients undergoing psychiatric treatment, patients with a history of heart, liver, kidney or other disease that might contraindicate corticosteroid therapy, patients who had undergone lung resection, patients undergoing treatment for tuberculosis or mycotic infections of the lungs and patients with tracheotomy or mechanical obstruction of the trachea. We also excluded patients with myopathies or neurological conditions (such as sequelae of stroke or encephalopathies) and patients with body mass index (BMI) > 40 kg/m^2^.

### Ethical matters

This study was approved by the Research Ethics Committee of UNIFESP (judgment number 0974/09; date: September 18, 2009). All patients provided written informed consent for participation, in accordance with international regulations for human subject research. When patients were underage (< 18 years of age), consent was obtained from their parents or legal guardians.

This study was registered in the Brazilian Registry of Clinical Trials (http://www.ensaiosclinicos.gov.br/) under accession number RBR-6XWC26; date: January 5, 2016.

### Sample

We studied 31 patients in the ciclesonide group and 27 patients in the hydrocortisone group. We calculated the sample size as prescribed by Greenberg,^22^ considering a FEV1 improvement of 0.37 ± 0.85 liters after intervention, thus resulting in 65 patients for each group.

### Study design

This was a double-blind, placebo-controlled, randomized clinical trial that was designed to compare the efficacy of inhaled ciclesonide versus intravenous hydrocortisone for managing moderate or severe acute asthma in an emergency department setting.

### Blinding

Both blinding and randomization were done centrally at the Neuro-Sono Sleep Center, São Paulo, Brazil. Blinding of active ingredients and their respective placebos was achieved by random allocation of four letters (A, B, C and D) to each of the following products: hydrocortisone, ciclesonide, hydrocortisone-identical placebo and ciclesonide-identical placebo. After random allocation of letters to designate each product, we defined two product pairs: Inhaled Active Ingredient + Intravenous Placebo; and Intravenous Active Ingredient + Inhaled Placebo. This was done in random combinations that enhanced the safety of blinding. Both the intravenous placebo and the inhaled placebo were identical to their active counterparts.

Information about the intervention that each patient randomized to the study would receive was distributed in opaque numbered envelopes, which were only opened at the time of use. The nursing staff prepared the medications for administration as instructed in the numbered envelopes. The staff who prepared the medications, the providers who administered them and all researchers involved were blinded to the active pharmaceutical ingredients of interest.

### Randomization

Patients included in the sample were recorded consecutively in a logbook and were assigned a serial number.

The 58 patients were divided into two groups: study (ciclesonide) and control (hydrocortisone), in accordance with two computer-generated random number tables. Each table contained an ascending sequence of numbers. Patients were allocated to one or the other according to the serial number attributed at the time of enrollment, which ensured that neither staff nor patients were aware of the intervention to which each patient would be allocated.

### Ciclesonide group

Patients received ciclesonide at a dose of 160 mcg/puff. The first dose was administered five minutes after inclusion in the trial, and consisted of three puffs (480 mcg); the second dose at 20 minutes (480 mcg); and the third dose at 40 minutes (480 mcg). Thus, the total dose was 1440 mcg. Patients in this group also received hydrocortisone-identical placebo at five minutes. Since there was no standard recommendation, we took the total dose of 1440 mcg to represent a high dose, in accordance with the Global Initiative for Asthma (GINA) (www.ginasthma.org). This also needed to be given during the first hour after admission to the emergency room.

### Hydrocortisone group

Patients in this group received 500 mg of hydrocortisone intravenously and ciclesonide-identical placebo at 5, 20 and 40 minutes.

### Both groups

Both groups received short-acting bronchodilators (fenoterol hydrobromide and ipratropium bromide) at 0, 10 and 30 minutes.

### Measurements

We used the spirometric variables FEV1 and peak expiratory flow (PEF) as primary outcome measurements, along with the clinical variables of dyspnea, wheezing and accessory muscle use during breathing (as assessed through observation of the sternocleidomastoid muscle). As secondary outcomes, we evaluated the heart rate, respiratory rate, blood pressure and pulse oximetry.

These parameters were measured every 30 minutes from the time of patient admission until the second hour and every 60 minutes thereafter until the fourth hour in the emergency department, thus making a total of six measurements. In this manner, we ensured rigorous monitoring throughout the patient observation period. We analyzed all six measurements, and no statistical difference was observed in comparisons of each paired time point (Table 1). For the purposes of this study, we analyzed and showed data from 30 minutes (baseline) and from the fourth hour, as we felt that these assessments were sufficient to represent the patients’ course, among the six measurements obtained.

**Table 1.** Comparison of spirometric variables between the two study treatments. Data from 30 minutes (baseline), 60 minutes, 90 minutes, 120 minutes, 180 minutes and 240 minutes

**FVC**												
**Time**	**30’**		**60’**		**90’**		**120’**		**180’**		**240’**	
	**C**	**H**	**C**	**H**	**C**	**H**	**C**	**H**	**C**	**H**	**C**	**H**
	2.82 ± 0.99	2.49 ± 0.80	2.93 ± 1.0	2.75 ± 0.88	2.95 ± 1.02	2.66 ± 0.83	2.96 ± 1.02	2.67 ± 0.77	2.90 ± 1.07	2.69 ± 0.79	2.83 ± 0.99	2.67 ± 0.81
**FEV1**												
**Time**	**30’**		**60’**		**90’**		**120’**		**180’**		**240’**	
	**C**	**H**	**C**	**H**	**C**	**H**	**C**	**H**	**C**	**H**	**C**	**H**
	1.80 ± 0.84	1.59 ± 0.68	1.87 ± 0.80	1.72 ± 0.64	1.93 ± 0.85	1.73 ± 0.65	1.91 ± 0.84	1.78 ± 0.63	1.89 ± 0.87	1.80 ± 0.64	1.82 ± 0.84	1.78 ± 0.61

FVC = forced vital capacity; FEV1 = forced expiratory volume in the first second; C = ciclesonide; H = hydrocortisone.

### Procedures

**Figure 1.**
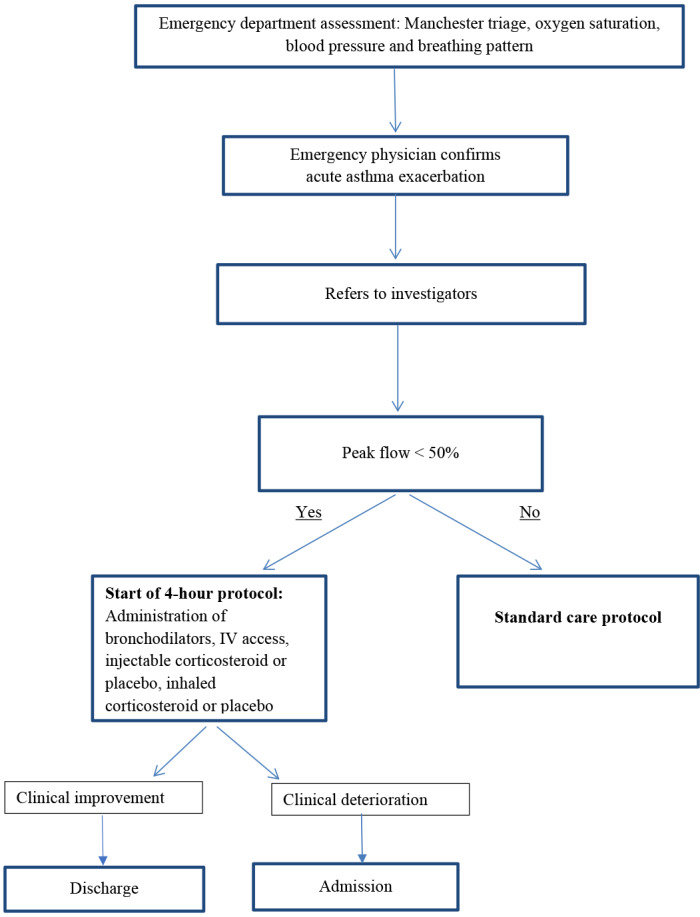
Flow diagram of patient inclusion and care.

The emergency room nurse applied the Manchester triage system and measured oxygen saturation, blood pressure and breathing pattern. The emergency room physician then confirmed the diagnosis of asthma exacerbation and notified the investigators, who performed an initial assessment by measuring peak flow and explained the study to the patient. Patients with a peak flow less than 50% of the predicted flow were invited to participate in the study (Figure 1), as the sample was designed to include only severe patients.

Once the patient had been included, the investigators worked with the emergency department staff to provide all the necessary care and perform the measurements required for the study.

Spirometric parameters were measured in an Easy One model 2009 spirometer (NDD Medizintechnik AG, Zurich, Switzerland). The best of three successive expiratory curves was considered valid and was used for the analysis, as recommended by the American Thoracic Society. Peak flow was estimated using the Mini-Wright Peak Flow meter (Clement Clarke, Hanlon, United Kingdom). Again, the highest of three measurements was used for the analysis.

Dyspnea was assessed subjectively as perceived shortness of breath, using the Borg scale: this is a visual analogue scale from 0 to 10, on which 0 represents absence of dyspnea and 10 is the maximum dyspnea. During the initial assessment and at each time point for reassessment, we evaluated wheezing and accessory muscle use. Wheezing was assessed through pulmonary auscultation and was ranked from 0 to 3 on an ascending scale of severity (0: no wheezing; 1: slight wheezing; 2: moderate wheezing; 3: severe wheezing). Accessory muscle use was also measured on a scale of increasing intensity (0: no accessory muscle activity: 1: slight activity; 2: moderate activity; 3: marked accessory muscle activity). When there was little wheezing or a silent chest plus marked accessory muscle use or signs of muscle fatigue, dyspnea was classified as severe. Individual and pooled analyses were performed for all parameters.

### Criteria for improvement

Patients were evaluated for improvement at all assessment time points, to ensure patient safety and detect any possible need for additional interventions other than those provided for in the study protocol. For the purposes of this study, we considered the following definitions of improvement: 1) FEV1 and PEF ≥ 70% of those predicted for the age, sex, weight and height; and 2) improvement of dyspnea: a) Borg score < 2;^23^ b) reduction of wheezing severity from baseline; and c) no accessory muscle use, as determined through observation of the sternocleidomastoid muscles.

### Interim analysis

We planned to conduct an interim analysis at the time when the number of patients included had reached approximately half the predicted sample size, in order to decide whether to continue or to terminate inclusion. This analysis was carried out at the randomization and blinding center (Neuro-Sono Sleep Center) by a committee that was established specifically for this purpose. After inclusion of 58 patients, this interim analysis committee suggested that recruitment for the study should be halted, since no difference between the treatments had been detected.

### Adverse events

We actively evaluated the more frequent adverse events, such as dry mouth, tremor, palpitations, anxiety and headache, and recorded any other patient-reported events.^24,25^ These variables were evaluated in terms of intention to treat (ITT).

### Statistical analysis

The sample size was calculated considering a change in FEV1 of 0.37 liters, after treatment, as an indicator of improvement; a standard deviation of 0.85 liters; a significance level of 5%; and a statistical power of 80%.^16,22^ This resulted in a sample size of n = 130 patients, i.e. 65 patients in each group. As noted above, interim analyses were carried out as planned after enrollment of 30 patients in each group; at that time, in view of the results, the interim analysis committee recommended termination of enrollment.

Quantitative variables were expressed as the mean ± SD, and categorical variables, as n (%). We used Student’s t test for independent samples for normally distributed data, the Mann-Whitney U test for asymmetrically distributed data and Pearson’s chi-square test or Fisher’s exact test for categorical data.^26,27^ Outcomes were assessed using ITT, considering the worst scenario, i.e. losses in the study group were considered treatment failures and losses in the control group, successful treatment. P-values < 0.05 were considered statistically significant.

### Availability of data and materials

All the data generated and analyzed during this study are available upon contact with authors.

## RESULTS

Thirty-one patients in the ciclesonide group and 27 patients in the hydrocortisone group were analyzed using ITT.

### Demographic data

The ciclesonide and hydrocortisone groups (Table 2) were similar with regard to age, systolic blood pressure (SBP), diastolic blood pressure (DBP) and the proportions of smoking, hypertension (HTN), diabetes mellitus (DM) and alcohol use. There were more women in the ciclesonide group (P < 0.001).

**Table 2.** Clinical and demographic characteristics of the ciclesonide and hydrocortisone groups

Variable	Group		P-value
	Ciclesonide (n = 31)	Hydrocortisone (n = 27)	
Sex: female, n (%)	23 (74)	18 (66)	< 0.001
Age (years), mean ± SD	38.3 ± 13.58	39.0 ± 18.99	0.826
BMI (kg/m^2^), mean ± SD	25.9 ± 5.75	28.0 ± 5.44	0.094
SBP (mmHg), mean ± SD	120 ± 14.27	127 ± 14.55	0.057
DBP (mmHg), mean ± SD	78 ± 12.34	75 ± 17.85	0.477
Smokers (current and former), n (%)	9 (29.0)	8 (30)	0.960
HTN, n (%)	3 (10)	6 (22.0)	0.175
DM, n (%)	0 (0.0)	0 (0.0)	0.180
Alcoholism, n (%)	1 (3.2)	1 (3.4)	0.920
Obesity, n (%)	7 (22.58)	10 (37.03)	0.727

BMI = body mass index; SBP = systolic blood pressure; DBP = diastolic blood pressure; HTN = hypertension; DM = diabetes mellitus; SD = standard deviation.

The ciclesonide and hydrocortisone groups did not differ regarding vital signs and pulse oximetry (Table 3). In within-group assessments, as expected, heart rate (HR) and respiratory rate (RR) were lower at hour 4, which was consistent with the clinical improvement observed in both groups. In between-group analyses, pulse oximetry and vital signs did not differ at hour 4.

**Table 3.** Response to treatments using hydrocortisone and ciclesonide, at hour 4, considering heart rate (HR), respiratory rate (RR), oxygen saturation (SpO2), systolic blood pressure (SBP) and diastolic blood pressure (DBP)

Variable	Ciclesonide (n = 31) (mean ± SD)	Hydrocortisone (n = 27) (mean ± SD)	P	Absolute effect size (95% CI)
HR	86 ± 23.14	90 ± 11.30	0.404	4.0 (-13.19; 5.19)
RR	18 ± 3.74	18 ± 5.52	0.679	0.0 (-2.46; 2.46)
SpO2	97 ± 3.19	95 ± 3.40	0.144	2.0 (0.30; 3.70)
SBP	120 ± 15.64	127 ± 14.32	0.092	7.0 (-14.71; 0.71)
DBP	75 ± 9.07	79 ± 12.31	0.208	4.0(-9.64; 1.64)

SD = standard deviation; CI = confidence interval.

### Clinical variables

All the clinical parameters evaluated in this study showed improvement at hour 4, compared with entry to the emergency room. There was also no difference between the effects of ciclesonide and those of hydrocortisone at hour 4, i.e. both treatments were equally effective in improving respiratory effort, accessory muscle use, wheezing and Borg dyspnea scale scores (Table 4).

**Table 4.** Effect of treatment on the variables of respiratory effort, accessory muscle use, wheezing and Borg dyspnea scale score at hour 4

Variable	Ciclesonide (n = 31)		Hydrocortisone (n = 27)		P	Relative effect size OR (95% CI)
	n (%)	Absolute effect size (95% CI)	n (%)	Absolute effect size (95% CI)		
Respiratory effort	1 (3)	32/1000	1(3)	28/1000	0.95	0.87 (0.05; 14.56)
Accessory muscle use	1 (3)	32/1000	2 (6)	28/1000	0.55	0.42 (0.04; 4.87)
Wheezing	8 (25)	258/1000	9 (31)	232/1000	0.55	0.70 (0.22; 2.16)
Borg ≥ 8	0 (0)	0/1000	0 (0)	0/1000	0.99	NE

SD = standard deviation; CI = confidence interval; OR = odds ratio; NE = not estimable.

### Spirometric variables

The patients treated with inhaled ciclesonide and those treated with hydrocortisone exhibited similar forced vital capacity (FVC), FEV1 and PEF values and similar progression of these parameters (Table 5). At hour 4, neither the FVC nor the FEV1 values had changed from baseline in either group. PEF increased significantly from 30 minutes (baseline) to hour 4 (P < 0.001) in both groups, and both treatments were equally effective when compared head-to-head at hour 4 (Table 6).

**Table 5.** Progression of spirometric variables from baseline (30 minutes) to hour 4 and comparison of the two study treatments at hour 4

Variable	Ciclesonide (Mean ± SD)		P	Hydrocortisone (Mean ± SD)		P	C x H Hour 4
	Baseline	Hour 4		Baseline	Hour 4		P
FVC	2.82 ± 0.99	2.83 ± 0.99	0.95	2.49 ± 0.80	2.67 ± 0.81	0.41	0.50
VEF1	1.80 ± 0.84	1.82 ± 0.84	0.94	1.59 ± 0.68	1.78 ± 0.61	0.28	0.83
PEF	157.58 ± 48.11	276.89 ± 100.46	< 0.001	170.18 ± 54.21	293.0 ± 92.24	< 0.001	0.51

SD = standard deviation; C x H = ciclesonide versus hydrocortisone; FVC = forced vital capacity; FEV1 = forced expiratory volume in the first second; PFE = peak expiratory flow.

**Table 6.** Comparison of the spirometric variables of the two study treatments at hour 4

Variable	Ciclesonide (n = 31) Mean ± SD	Hydrocortisone (n = 27) Mean ± SD	P	Absolute effect size (95% CI)
FVC	2.83 ± 0.99	2.67 ± 0.81	0.50	0.16 (-0.30; 0.62)
FEV1	1.82 ± 0.84	1.78 ± 0.61	0.83	0.04 (-0.30; 0.41)
PEF	276.89 ± 100.46	293.0 ± 92.24	0.51	-16,11 (-65.72; 33.50)

SD = standard deviation; CI = confidence interval; FVC = forced vital capacity; FEV1 = forced expiratory volume in the first second; PFE = peak expiratory flow.

### Adverse events

More patients in the hydrocortisone group complained of dry mouth, but there was no statistically significant difference in the frequency of any adverse effect between the groups (Table 7).

**Table 7.** Adverse events in the two groups

Event	Ciclesonide (n = 31)			Hydrocortisone (n = 27)		
	n	%	95% CI	n	%	95% CI
Dry mouth	2	6.5	0 to 15.0	7	25.9	12.0 to 39.9
Palpitations	3	9.7	0 to 15.0	1	3.7	0 to 10.8
Tremors	11	35.5	0 to 37.4	7	25.9	12.0 to 39.6
Headache	2	6.5	0 to 15.0	3	11.1	10.4 to 11.8
Anxiety	1	3.2	3.0 to 3.4	2	7.4	0 to 17.3

CI = confidence interval. There was no significant difference between groups; dry mouth was the only complaint that was more prevalent in the hydrocortisone group.

### Hospitalization, losses and exclusions

Two patients in the ciclesonide group developed worsening bronchospasm and severe desaturation early in the course of treatment (having received only one dose of medication), and ultimately required ventilatory support.

## DISCUSSION

To the best of our knowledge, this was the first double-blind randomized clinical trial to test high-dose inhaled ciclesonide for managing acute asthma in the emergency department. Our findings suggest that high-dose inhaled ciclesonide is as effective as intravenous hydrocortisone for this purpose. In this study, we tested ciclesonide as the intervention because it is a prodrug with high potency and less potential for oropharyngeal side effects than inhaled corticosteroid. This is particularly important for use in situations of acute exacerbations of asthma, a setting in which high doses of inhaled corticosteroids need to be administered.^12^

Studies have shown that use of inhaled and systemic corticosteroids can decrease the length of emergency department stay and the hospitalization rate, when administered in the first hour of an acute asthma exacerbation.^16^ Nevertheless, the optimal agent, dosage and duration of observation in the emergency department remain unknown.^20,28^

Both drugs reduced expiratory effort, wheezing and accessory muscle use (Table 4); however, among the spirometric parameters analyzed, only PEF improved significantly from baseline at hour 4 in both groups (Table 6). Adverse events, such as dry mouth, palpitations, tremor, headache and anxiety, did not differ between the two groups (Table 7).

Clinical studies using high doses of inhaled corticosteroids such as fluticasone,^16^ flunisolide^17^ and ciclesonide^11^ also found these agents to be effective in increasing peak flow.

Although we did not enroll a large number of patients, the groups did not differ in terms of demographic characteristics, except for the higher proportion of women in the ciclesonide group (Table 2). Two patients in the ciclesonide group, both with peak flow < 30% of the predicted flow, developed worsening bronchospasm and severe desaturation early in the course of treatment (having received only one dose of medication), and ultimately required invasive ventilation. Given the small sample, the likelihood of between-group differences was very high, and we judged these events to be attributable to chance.

Clinical parameters (Table 4) and vital signs (Table 3) were similar at admission to the emergency room and at hour 4. Only DBP was higher in the hydrocortisone group, possibly due to the systemic effects of the corticosteroid.^3^

FVC and FEV1 remained unchanged from baseline to hour 4, and did not differ between the two groups. In previous studies on fluticasone^16^ and flunisolide,^17^ improvements in these parameters were reported. In our study, we observed an increase in PEF despite no increase in FEV1. This is consistent with the well-known mismatch between FEV1 and PEF in cases of acute severe asthma,^29-31^ a condition in which FEV1 is underestimated and does not correlate adequately with rises in peak flow.

In a Cochrane review, it was noted that the higher cost of inhaled corticosteroids, compared with systemic corticosteroids, was an obstacle to use of the former.^28^ However, this was not an issue in our study, in which nine puffs of ciclesonide (the total dose used in the emergency department) had an estimated cost of US$ 2.47, while a single 500-mg dose of hydrocortisone had a cost of US$ 3.18, thus making ciclesonide more cost-effective. In the United States, the average cost of treatment reaches US$ 1368.00, 30 days after a severe asthmatic exacerbation.^32^ Also with regard to the cost and utility of inhaled corticosteroids, the FourFold Asthma Study (FAST) showed that it is clinically safe for patients to simply quadruple their usual dose of inhaled corticosteroids at home, upon deterioration of their condition, thus aborting a severe asthma attack and obviating the need for hospitalization.^33^

The limitations of our study included the lack of follow-up (to assess for recurrence) and the small sample size. The latter had the consequence of, for instance, preventing us from determining whether dry mouth was truly more prevalent in the hydrocortisone group. The strengths of our study included its design and external validity, since we included adult patients from the general population with no restrictions regarding age, gender or ethnic group; rigorous evaluation of clinical and spirometric parameters; appropriate masking and blinding; and rigorous close monitoring of patients for a four-hour period during the study protocol.

## CONCLUSION

In summary, our study suggests that high-dose inhaled ciclesonide is as effective as injectable hydrocortisone for managing acute severe asthma and had a similarly favorable adverse-event profile, with the advantage of being a prodrug that exerts topical anti-inflammatory effects while reducing the risk of long-term systemic side effects.
